# Reporting the characteristics of the policy context for population-level alcohol interventions: A proposed ‘Transparent Reporting of Alcohol Intervention ContExts’ (TRAICE) checklist

**DOI:** 10.1111/dar.12201

**Published:** 2014-10-01

**Authors:** John Holmes, Petra S Meier, Andrew Booth, Alan Brennan

**Affiliations:** 1 School of Health and Related Research, University of SheffieldSheffield, UK

**Keywords:** realist synthesis, policy transfer, policy evaluation, policy context, alcohol policy

## Abstract

**Issues:**

Effectiveness of alcohol policy interventions varies across times and places. The circumstances under which effective polices can be successfully transferred between contexts are typically unexplored with little attention given to developing reporting requirements that would facilitate systematic investigation.

**Approach:**

Using purposive sampling and expert elicitation methods, we identified context-related factors impacting on the effectiveness of population-level alcohol policies. We then drew on previous characterisations of alcohol policy contexts and methodological-reporting checklists to design a new checklist for reporting contextual information in evaluation studies.

**Key Findings:**

Six context factor domains were identified: (i) baseline alcohol consumption, norms and harm rates; (ii) baseline affordability and availability; (iii) social, microeconomic and demographic contexts; (iv) macroeconomic context; (v) market context; and (vi) wider policy, political and media context. The checklist specifies information, typically available in national or international reports, to be reported in each domain.

**Implications:**

The checklist can facilitate evidence synthesis by providing: (i) a mechanism for systematic and more consistent reporting of contextual data for meta-regression and realist evaluations; (ii) information for policy-makers on differences between their context and contexts of evaluations; and (iii) an evidence base for adjusting prospective policy simulation models to account for policy context.

**Conclusions:**

Our proposed checklist provides a tool for gaining better understanding of the influence of policy context on intervention effectiveness. Further work is required to rationalise and aggregate checklists across interventions types to make such checklists practical for use by journals and to improve reporting of important qualitative contextual data. *[Holmes J, Meier PS, Booth A, Brennan A. Reporting the characteristics of the policy context for population-level alcohol interventions: A proposed ‘Transparent Reporting of Alcohol Intervention ContExts’ (TRAICE) checklist.* Drug Alcohol Rev*2014;33:596–603]*

## Introduction

The effectiveness of alcohol policy interventions is rarely consistent across different times and places. Robin Room made a valuable contribution to how we think about heterogeneity in policy evaluation evidence when discussing unexpected null effects on alcohol consumption following alcohol affordability increases in the Nordic countries 1–3. To better understand these findings, Robin and his colleagues drew on his deep knowledge of the alcohol literature across multiple disciplines to provide a typically lucid and detailed description of how characteristics of the wider policy context exert upwards, downwards or stabilising pressures on the alcohol consumption trend. Robin then set a challenge for the next generation of research by calling for greater consideration of these context factors when assessing policy evaluation results so as to improve understanding of when and how interventions are effective [4]. We take steps towards responding to this challenge by presenting a proposed ‘Transparent Reporting of Alcohol Intervention ContExts’ (TRAICE) checklist for reporting of contextual information within population-level alcohol policy evaluation studies and discussing the role of such a checklist in evidence synthesis and policy-making processes.

Concerns about the applicability of evidence to different contexts are partly driven by the increasing importance of ‘policy transfers’ over recent decades [5],[6]. The facility to affect policy transfer is fundamental to evidence-based policy-making as it relates to processes by which knowledge pertaining to the development, implementation, operation and effectiveness of policies in one context is used to inform policy decision-making elsewhere. Within health research, analyses of the transferability of evidence have focused on pharmacological, community or individual-level interventions. These studies concluded there is inadequate reporting of intervention components, adaptability and fidelity in primary research 7–11 and the ‘Template for Intervention Description and Regulation’ has been developed partly to address these concerns [12]. Less attention has been given to population-level interventions where large differences between policy contexts may be seen when comparing evaluations. This is significant as the case study literature on policy transfers indicates that success is dependent not only on policy-makers' understanding and responding appropriately to the components of interventions and the mechanisms by which these lead to desired effects, but also the equally important interaction between policy components and mechanisms on the one hand and the context into which the policy is implemented on the other [5].

Room. argued that this interplay with policy context is often reduced to simple invocations of *ceteris paribus* [4], for example in meta-analyses. Although statistically valid, such statements are of little practical value when differences between contexts are key determinants of policy effectiveness and successful policy transfer. Contextual specificity of meta-analyses may be improved via meta-regression techniques that adjust effectiveness estimates for confounding context factors [13] and clear reporting of contextual data would facilitate this. It would not typically be possible to include all relevant factors, but systematic identification of factors and selection of the most pertinent would still advance understanding of policy effectiveness. An alternative approach is qualitatively driven narrative or process evaluation where detailed examination of how and why a policy operates within its context is undertaken. This often affords greater understanding of contextual influences [14]; however, external validity is often constrained by the small number of contexts examined. A third approach, ‘Realist evaluation’, addresses this limitation by seeking to combine detailed and contextualised analyses with systematic validation of findings across multiple evaluations [15]. In practice, it provides a method that aligns well with the principles of successful policy transfer and argues that researchers must: (i) identify the policy components and mechanisms through which effects are produced; (ii) examine evaluations of the policy to identify candidate context factors that may impact on these processes; (iii) use existing evidence and theory to hypothesise mechanisms by which this contextual impact occurs; and (iv) use future evaluations to test and refine understandings of which context factors are important and what mechanisms allow them to shape policy effectiveness [16],[17].

Despite the value of contextual data to successful policy transfer and evidence synthesis methods, there has been no established guidance for authors on reporting research context. Therefore, we propose the TRAICE checklist to address this gap. Recognising that a ‘one-size fits all’ checklist is not feasible due to the requisite information varying across interventions, we nevertheless believe some aggregation is possible. We thus designed TRAICE for reporting on evaluations of population-level alcohol policy interventions. We particularly focused on interventions restricting the availability or affordability of alcohol as this is where much of the population-level alcohol policy evidence lies [18].

## Methods

The mechanisms by which population-level alcohol policies impact on alcohol consumption and related harms are well documented [18],[19]. For example, pricing policies seek to suppress demand by increasing prices and reducing affordability such that reductions in consumption and harm follow. Therefore, the key elements of checklist development were to identify the domains to be included in the checklist (context factors and the hypothesised mechanisms by which factors impact on policy effectiveness), to analyse and review the potential list and refine it into a checklist, and then to specify the data sources from where the relevant information can be obtained.

Room. have not provided a method for identifying context factors [4] so instead we drew on methods used in the wider health sciences literature (e.g. reviewing primary studies and reviews for factors included in statistical analyses, expert elicitation or panel discussion) on context factors affecting different intervention types and describe these below [9],[11],[20]. As a pragmatic consideration, we only sought factors hypothesised to have a direct impact on policy effectiveness (i.e. where at least part of the impact was not mediated by other factors) and excluded distal factors where impacts were only indirect (see Holder for a discussion of these [21]).

Our initial intention was to harness systematic review techniques and search publication databases (PubMed, PsychInfo, CINAHL and Web of Knowledge) for studies that specifically consider the impact of one or more context factors on intervention effectiveness. Search strings included variants of the terms ‘alcohol*, liquor* and drink*’ combined with terms relating to ‘policy’ and ‘context*’, ‘setting’ or ‘environment*’. This initial scoping search revealed that the concept of context is typically absent from titles and abstracts although three articles using regression-based methods to quantify the effects on alcohol price elasticities of the following context factors were identified: time period, baseline consumption, retail licensing systems and population-level beverage preferences 22–24. As our systematic review approach was unsuccessful, we focused on two methods to identify context factors.

Although few studies had specifically researched context factors, we noted many papers mentioned context in discussion sections when constructing explanations for study findings. Therefore, we analysed a purposive sample of 112 papers included in a recent review of alcohol price elasticities [25]. Although pricing policies are just one example of a population-level intervention, many context factors identified will have relevance to population-level interventions in general. Two researchers examined each paper's discussion section and used qualitative synthesis methods [26] to extract relevant passages, code these thematically and identify an initial set of context factors from the coding frame alongside hypothesised mechanisms of effect described in the associated text. The research team then identified potential redundancies and overlaps across factors resulting in some being merged prior to agreement on a conclusive set of factors. To complement this analysis, we asked eight members of our expert international advisory group to comment on the identified context factors and mechanisms. The ensuing discussion critiqued the completeness and appropriateness of our results with factors added or removed accordingly. To aid design and conciseness of the checklist, experts were also asked to consider the relative importance of factors, whether impacts were direct or indirect, whether proposed mechanisms were supported by theory or empirical evidence and whether alternative hypothesised mechanisms could be justified.

A further consideration was whether TRAICE should address all or only some of the identified context factors as this linked to issues of data accessibility, burden on authors and ability to standardise reporting. To develop TRAICE, the lead author produced an initial draft that sought to reconcile identified context factors with considerations of feasibility. This draft was then reviewed and revised by members of the research team in an iterative process analogous to that used when developing STROBE, CONSORT and PRISMA.

The following step, decisions on the specific data to be recorded, was informed by documents with similar aims, such as the International Alcohol Control Study's ‘Alcohol Environment Protocol’ [27] and the World Health Organization Global Status Report on alcohol and health [28].

For the final design of the checklist, we sought to follow the format of established reporting checklists (e.g. PRISMA, STROBE and CONSORT 29–31). These checklists seek to balance conciseness with detail. Each contains approximately 25 items to ensure reporting is comprehensive without imposing impractical burdens on authors. They prompt authors to identify where in their report (the page number) required information is provided. They mix required information with suggestions for further detail, if relevant. They also offer supporting information to aid checklist completion. We attempted to follow this structure while recognising that as TRAICE seeks to elicit a large amount of supplementary information to interpret findings, and this information may not be of interest to general readerships, some contextual detail may be best placed in appendices rather than integrated into the main report.

## Results

**Figure 1:**
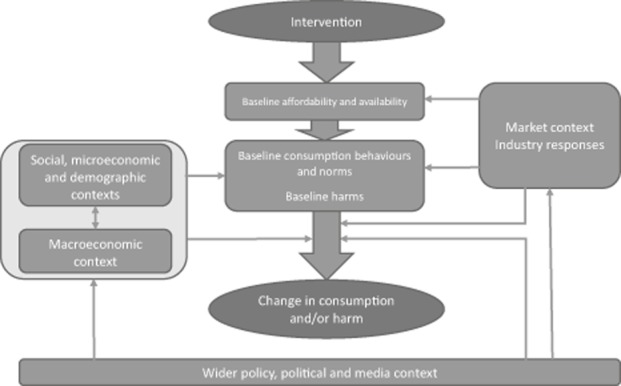
Conceptual map of relationships between context factors influencing population-level alcohol policy effectiveness.

The final list of context factors cover six broad contextual domains as detailed in Table 1. These were then converted into actual checklist items as shown in Table 2. For reasons of space, this paper focuses on the checklist rather than describing each individual context factor and the mechanisms of effect that are largely discussed elsewhere [4],[21]. However, we mapped the identified domains and hypothesised mechanisms in Figure 1 together with a brief description to aid understanding.

**Table 1:** Identified context factors influencing population-level alcohol policy effectiveness

Baseline consumption and harm	Social, microeconomic and demographic context	Macroeconomic context
Mean consumption Dominant drinking patterns Distribution of consumption Beverage preferences Drinking location preferences Alcohol-related social norms Cultural position of alcohol Baseline rates of alcohol-related harms	Age distribution Disposable income distribution Ethnic composition of population Religiosity of population	Economic growth rate Unemployment rate Inflation rate
Baseline conditions in intervention area	Market context and industry responses	Wider policy, political and media context
Baseline affordability Baseline availability	Diversity of the market Availability of alternative markets Competitiveness of the market Industry responses to intervention	Wider alcohol policies Wider public policies Media and political discussion of alcohol problems

**Table 2:** Proposed checklist for reporting contextual data in population-level alcohol policy evaluation reports

	Baseline consumption and harm^a^	Page no.
1	State baseline abstinence and per capita consumption levels and describe recent consumption trends in these variables.	
2	State how per capita consumption is distributed across the research setting's main beverage categories (e.g. beer, wine, spirits) and describe recent trends in this distribution.	
3	State the WHO pattern of drinking score for the country in which the research is set.	
4	State baseline rates of alcohol-related harms for any harm used in the analysis and for the following additional categories: (i) liver disease; (ii) road traffic accidents; and (iii) alcohol use disorders. Describe recent trends in these harms.	
5	Give consideration to and report on how any of the following may have influenced the results: (i) trends in alcohol consumption or alcohol-related harm (see items 1–4) within demographic subgroups of the population (e.g. gender, age and socioeconomic groups), (ii) drinking location preferences, (iii) the cultural position of alcohol; and (iv) alcohol-related social norms and practices.Where possible quantitative data should be included. These may be found in behavioural or attitudinal surveys detailing, for example, whether alcohol is routinely consumed with meals, whether drinking to intoxication is viewed as acceptable, and other attitudinal data relating to drinking or particular patterns of drinking.	
	Baseline conditions in intervention area	
6	For the drink(s) categories and location(s) preferred by heavy drinkers, state how much a typical drink serving costs and indicate how this compares with the average hourly wage earned by an unskilled worker.	
7	State the number of off-sales and on-sales premises per capita adult (16+) and indicate if there are large ‘dry areas’ in the research setting (e.g. local prohibition areas or areas with substantially lower alcohol availability for reasons other than urbanity).	
	Social, microeconomic and demographic context^b^	
8	State the proportion of the population in the following age groups: 0–15, 16–24, 25–39, 40–59, 60–74, 75+.	
9	State the poverty rate (with definition) and gini coefficient for the research setting.	
10	State the proportion of the population in major ethnic groups.	
11	State the proportion of the population identifying themselves as belonging to major religions.	
12	Provide information on any relevant trends in the above factors.	
	Macroeconomic context^c^	
13	State the economic growth rate during the study period and note any periods of economic recession.	
14	State the baseline unemployment rate, note any substantive trend in unemployment and identify any relevant population groups with high unemployment rates.	
15	Identify any relevant groups at particularly high unemployment risk.	
16	State the inflation rate during the study period.	
	Market context and industry responses^d^	
17	State the proportion of total alcohol consumption, which is unrecorded consumption.	
18	State whether the alcohol market is a full or partial state monopoly or wholly privatised and describe the nature of any monopoly.	
19	Describe any noteworthy responses by producers or retailers to the intervention	
	Wider alcohol policy, political and media context	
20	Summarise the alcohol policy context in the research setting using the WHO Global Status Report format.	
21	Identify other alcohol policies introduced prior to or during the intervention period that may have affected evaluation results.	
22	Identify non-alcohol public policies introduced prior to or during the intervention period that may have affected evaluation results.	
23	Describe the tone and level of political and media debate on alcohol-related problems prior to and during the intervention period. Where possible, quantitative data should be included. These may found in studies or datasets quantifying the number of media articles on a given topic over a time period, broad content analysis of a sample of articles (e.g. whether the policy is portrayed positively or negatively) and stating whether the intervention was presented by government as being required for fiscal, public health or law and order (or other) purposes.	

^a^Provide the most up-to-date figures available. Figures for 2005/2006 are available for most countries in the WHO Global Status Report on Alcohol and Health 2011. ^b^Data sources for this section include national censuses or statistics data and World Bank microeconomic data. ^c^Contemporary and historical economic data are available from the World Bank data bank. ^d^Unrecorded consumption estimates for 2005/2006 are available for most countries in the WHO Global Status Report on Alcohol and Health 2011. WHO, World Health Organization.

At the centre of Figure 1 is the relationship between: (i) the intervention; (ii) baseline affordability and availability; (iii) baseline consumption behaviours or norms and baseline harms; and (iv) changes in consumption and harm. These represent the key policy components and their mechanisms of effect. For example, baseline conditions are likely to play an important role in determining policy outcomes. If affordability is already high, such that demand is largely saturated, increases in affordability will likely produce smaller effects than in situations where demand is partially suppressed [32],[33]. The other contextual domains interact with these baseline conditions and also with the mechanisms by which the policy takes effect. For example, different age, gender and socioeconomic groups may, firstly, have different baseline consumption behaviours and norms and, secondly, their responsiveness to price changes may be independent of these baseline behaviours and be related to their varying economic resources and spending priorities [34,35]. Similarly, characteristics of the market context, including opportunities to substitute with cheaper products, availability of illicit or cross-border markets and the extent of promotional activity will shape the baseline conditions at the centre of Figure 1, but also influence how drinkers respond to price changes by restricting or increasing the options available [32,36–38].

Table 2 presents the proposed TRAICE checklist for reporting of contextual information. It contains 23 items divided into the six domains in Figure 1. The design of TRAICE drew extensively on the World Health Organization Global Status Report on alcohol and health [28], which provides information on context factors for most countries. In particular the report provides information on beverage-specific alcohol consumption trends, illicit alcohol consumption, gender-specific rates for abstention, heavy episodic drinking, alcohol use disorders and dependence, liver cirrhosis and road traffic accident mortality, and characterisation of the alcohol policy context. As such, it provides a minimum reporting standard for consumption, harm and alcohol policy context data to which authors can turn in the absence of more up-to-date information. For factors not covered by the World Health Organization report, TRAICE generally focuses on readily accessible data (e.g. population demographics, standard macroeconomic indicators and basic policy structures) and data sources.

In general, the checklist prioritises recording of quantitative data and places less weight on descriptions of less tangible factors (e.g. alcohol-related social norms). Although intangible factors are important, it is more challenging to standardise reporting for such factors. Instead, authors are encouraged to consider, and to provide additional information on, areas of specific relevance to interpretation of results (e.g. describing the tone and level of media debate or noteworthy industry responses to an intervention). Examples of ways these may be provided in quantitative form are given, although we acknowledge this may often not be possible. Authors are also encouraged to identify and describe relevant trends in key factors. For these aspects, TRAICE serves more as an *aide memoire* to good practice rather than a prescriptive document.

## Discussion

Efforts to improve the use of scientific evidence in policy decision making rely in part on policy actors understanding the validity of evidence to their specific context. The proposed TRAICE checklist facilitates population-level alcohol policies by requiring that reports of evaluation studies provide research users with a more complete view of the context within which the evidence has been generated.

The development process for TRAICE was strengthened by the use of multiple methods to identify context factors. The methods were similar to those used in other studies examining the contextual specificity of evidence [9],[11],[20] and the results aligned well with previous examinations of the factors influencing changes in alcohol consumption and related harms. For example, the baseline affordability of alcohol (or consumer purchasing power), the alcohol market context and baseline drinking practices were consistently identified as important factors across our work and previous studies [4],[21]. However, the identification approach had significant limitations that we discussed in the Methods section above, and similar problems of inadequate reporting and non-consideration of contextual effects have been noted in other studies [7],[9],[10]. For pragmatic reasons, we did not seek to identify context factors not impacting directly on policy effectiveness. Holder presented an analysis of the policy context using a systems approach that incorporates more detail and a more complex model of the links between factors [21]. However, completion of a more exhaustive checklist based on this broader concept of the policy context would likely impose too great a burden on authors with respect to sourcing the necessary information. Our review of 112 studies identified a large number of context factors for alcohol price changes and these appear to have more general application as a list of potential influences on the effectiveness of other population-level policies. From this research, we have identified two priorities for further development of TRAICE: first, validation of our core list for non-pricing interventions by using wider data sources and multiple methods of data collection and, second, recognition that development of reporting checklists is a science in itself and, therefore, TRAICE should, in due course, be subject to a more formal development process of the kind recommended by Moher. and particularly involving a formal expert consensus process [39].

One strength of TRAICE lies in it being relatively straightforward to complete for national-level evaluations with most information available in standard national statistical outputs or international reports. However, this simplicity is achieved by privileging measurable context factors over important but less tangible factors. As previously explained, this pragmatic decision sought to reduce burden on researchers and to facilitate standardisation. We welcome suggestions for how systematic reporting of less tangible contextual information may be achieved and encourage development of measures to facilitate this. It is desirable that reporting checklists minimise burden on researchers where possible and an obvious solution is to shorten the checklist. To do this requires an understanding of which items are most important and we welcome feedback and evidence in this area. TRAICE may be more difficult to complete for sub-national evaluations where data are less available and reporting national-level data may be a necessary compromise under such circumstances. An additional limitation is that TRAICE functions best when applied to evaluations conducted over short contemporary timeframes where the context is stable and data characterising it are readily available. For evaluations utilising long-term time series data covering a continually changing policy context, recording of contextual data may be more challenging and, again, we invite suggestions to facilitate a more nuanced use of contextual data.

The TRAICE checklist can be used in three specific ways to further evidence-based policy-making. First, explicit and standardised recording of contextual data in future studies allows evidence synthesis techniques to exploit these data. For meta-analyses, each primary study would contain data on a set of variables potentially confounding estimates of policy effectiveness. These data could be easily extracted and entered into analytical software for use in meta-regressions with the independent effect of each context factor estimated within a meta-regression. Realist syntheses would also benefit as data would be readily available to allow the development and testing of hypotheses regarding impacts of context on policy effectiveness. Second, policy-makers assessing whether to undertake a policy transfer will have easier access to information on the salient differences between the research context and their own context. Although the policy process should not be mistaken for an exercise in scientific rationalism [40] and acknowledgement should be given to the role of policy makers' worldviews and values in informing their judgements on the validity of evidence [41], evidence syntheses informed by contextual data can improve the interface between decision-making processes and evidence. Third, simulation models appraising the potential effectiveness of future policies often rely on prior estimates of policy effectiveness as model inputs. Meta-regression results may be used to adjust those inputs to be more applicable to the modelled context or to test the sensitivity of results to alternative baseline conditions of interest.

These potential gains are contingent on scientific journals adopting requirements for reporting of contextual data. Although TRAICE represents a step towards that goal, further work is required beyond resolving those methodological issues identified above. A key challenge is that TRAICE addresses only population-level alcohol policy interventions. Checklists for community-and individual-level interventions as well as interventions in other areas of public health are needed; however, journals cannot be expected to compile compendia of checklists for all possible intervention types. Rationalisation of checklists across topics and interventions is required without sacrificing the understanding of interplay between policy components, mechanisms and contexts that drives the endeavour. From an author's perspective, one approach may be to develop an online decision-tree that maps to the intervention checklist required, thus presenting authors with an easy-to-follow route to the correct checklist. We encourage the research community, and particularly those with an interest an evidence-based policy-making, to comment on our proposed checklist and collaborate to further efforts in these areas.

## Conclusion

Successful policy transfer from a study context to an implementation context can be facilitated by an improved understanding of similarities and differences in contexts across both time and place. Our proposed TRAICE checklist of context factors influencing population-level alcohol policy effectiveness illustrates how such an understanding might be gained. It offers potential for facilitating meta-regression and realist synthesis and also presents a route to ensuring that policy-makers have easy access to relevant contextual information when judging the transferability of evidence to their context.

## Funding

Research reported in this paper was funded by the Medical Research Council and Economic and Social Research Council (Grant G100043).
